# Effect of storage, washing, and cooking on the stability of five pesticides in edible fungi of *Agaricus bisporus*: A degradation kinetic study

**DOI:** 10.1002/fsn3.1261

**Published:** 2019-11-12

**Authors:** Ali Heshmati, Mina Hamidi, Amir Nili‐Ahmadabadi

**Affiliations:** ^1^ Department of Nutrition and Food Safety School of Medicine Nutrition Health Research Center Hamadan University of Medical Sciences Hamadan Iran; ^2^ Medicinal Plants and Natural Products Research Center Hamadan University of Medical Sciences Hamadan Iran; ^3^ Department of Pharmacology and Toxicology School of Pharmacy Hamadan University of Medical Sciences Hamadan Iran

**Keywords:** *Agaricus bisporus*, Food processing methods, Gas chromatography/mass spectrometry, Mushroom, Pesticide residue

## Abstract

Pesticide residue in food products is one of the most important global health challenges. The current study sought to investigate the changes in pesticides residue levels in *Agaricus bisporus* under different storage conditions and during washing and cooking. Pesticides analysis was performed using gas chromatography/mass spectrometry (GC‐MS). The results showed that the half‐life (t_1/2_) of all of the studied pesticides stored at room temperature was lower than refrigerator and freezer temperature. In addition, the greatest reduction of diazinon, malathion, permethrin, propargite, and fenpropathrin was found at a pH of 12, 2, 12, 7, and 9, respectively. Although sodium chloride had no effective impact on pesticide reduction during the same washing times, the removal of pesticides increased as washing time increased. Further, the reduction of pesticides was time‐dependent during the boiling, microwaving, and frying processes. Based on these findings, the stability of insecticides, such as permethrin, malathion, and diazinon, was lower than acaricides, including propargite and fenpropathrin, in various techniques. Therefore, the use of washing solutions with an appropriate pH as well as increased cooking time may reduce the risk of pesticide exposure.

## Graphical abstract

The impact of storage, washing, and cooking on pesticides in Agaricus bisporus was studied. The storage in room temperature had the highest impact on pesticide dissipation. The pesticide reduction depended on its PKa and washing time and solution pH. The pesticides removal wasn't related to sodium chloride level in solution. The processing can be useful in reducing the risks of exposure with pesticides.

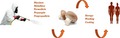

## 1 INTRODUCTION

Exposure to pesticide residues in foodstuff is one of the most important health challenges worldwide (Oliva, Cermeno, Camara, Martinez, & Barba, 2017; Zhang, Jiang, & Ou, 2011). Pesticides are used in the production of approximately one‐third of agricultural products. Without pesticide use, the loss of fruit, cereal, and vegetable yields from pest damage may reach 78%, 32%, and 54%, respectively (Zhang et al., 2011). The application of pesticides is expected to increase due to an expansion in the food supply to meet the world's growing demands (Reddy & Kim, 2015). It is predicted that the use of pesticides will be increased at least two to three times by 2020 in comparison with the past two decades (Nili‐Ahmadabadi et al., 2018). Of the millions of tons of pesticides used in agriculture, only 5% reach the target organism; the remainder affect other organisms or enter the water, soil and atmosphere (Kazemi, Tahmasbi, Valizadeh, Naserian, & Soni, 2012). This issue is particularly prevalent in developing countries, such as Iran.

After harvest, the storage conditions of food products may differ. In addition, they are often exposed to a variety of processing methods, such as washing, peeling, cooking, etc., which may affect pesticide residue, altering the level of humans’ exposure to these compounds from food (Heshmati & Nazemi, 2018; Savi, Piacentini, & Scussel, 2015; Wang et al., 2014). Therefore, its necessary to investigate the effects of applied processing in food industry on pesticide residue.

Pesticide residues have been reported in edible fungi (Barnes, Startin, Thorpe, Reynolds, & Fussell, 1995; Chang et al., 2014; Kamal et al., 2009; Xia, Tao, Yao, Wang, & Tang, 2016), which are one of the most important sources of nutrients in the world. In recent years, the per capita consumption of these products was approximately 4 kg (Koutrotsios, Kalogeropoulos, Kaliora, & Zervakis, 2018). *Agaricus bisporus* is one of the highest cultivated of edible fungi (Reis, Barros, Martins, & Ferreira, 2012). It contains significant amounts of protein, carbohydrates, and edible fiber and essential elements such as sodium, potassium, phosphorus, calcium, manganese, zinc, copper, vitamins, phenolic compounds and sterols. In addition, it also has antioxidant, antitumor, antiviral, hypocholesterolemic and hypoglycemic properties (Cheung, 2010; Heleno et al., 2015; Kalač, 2009; Wang et al., 2014).

Edible fungi are processed before consumption, but limited evidence on the role of these processes on the stability of pesticide residues in these products exists. In addition, no report has been published to simultaneously estimate the dissipation behavior of pesticides, such as diazinon, malathion, permethrin, propargite, and fenpropathrin, in *Agaricus bisporus* during various food processing methods. Therefore, in this study, the changes in these pesticide residues were investigated during the storage, washing, and cooking of *Agaricus bisporus*.

## 2 MATERIALS AND METHODS

### 2.1 Materials

All chemicals were obtained from Merck, Darmstadt, Germany, unless otherwise stated. Diazinon, malathion, permethrin, propargite, fenpropathrin standards (Purity 99%) and the primary secondary amine (PSA) were purchased from Supelco (Bellefonte, USA). The commercial pesticides were obtained from the local pesticide production companies, Tehran, Iran.

### 2.2 Sample preparation

The *Agaricus bisporus* samples were randomly collected from a local market in Hamadan City. The initial concentrations of diazinon, malathion, permethrin, propargite and fenpropathrin were determined. Then, the *Agaricus bisporus* samples were immersed in an aqueous solution of 1% v/v commercial pesticides for 20 min. Finally, the studies were performed on samples containing pesticides in various conditions.

### 2.3 The *Agaricus bisporus* samples’ storage conditions

For 10 days, the samples were stored at three different temperatures: room (25°C), refrigerator (4°C), and freezer (−18°C). Then, on days 2, 4, 6, 8, and 10, the pesticide residue was measured. The degradation kinetics of each pesticide in the *Agaricus bisporus* samples were calculated by plotting the residue concentrations against the samples’ storage times for each storage condition. To obtain the best‐fit curves and the maximum squares of the correlation coefficients for the pesticides under different conditions, the exponential relations and first‐order rate equation were determined. The stability of each pesticide was generally presented as its half‐life (t_1/2_) (i.e., the time it took for the pesticide concentration to reach 50% of its initial concentration). The degradation rate was calculated using the following first‐order equation: C_t_ = C_0_^e‐kt^, where C_t_ indicated the level of the pesticide residue (mg/kg) at a defined time (days), C_0_ indicated the initial pesticide concentration (mg/kg), and k, which was independent of C_t_ and C_0_, was the first‐order rate constant (per day). The half‐life (t_1/2_) was obtained from the k value of each experiment t_1/2_ = ln2/k (Jankowska, Kaczynski, Hrynko, & Lozowicka, 2016; Liang, Li, Li, Wu, & Liu, 2012).

### 2.4 The washing process of *Agaricus bisporus* samples

The samples were exposed to various washing conditions, including water washing at pH 2, 5, 7, 9, and 12 and saline solutions with various concentrations of 0, 0.1, 1, and 10% sodium chloride. The samples (50 g) were immersed for 10, 20, and 30 min in washing solution and then rinsed with water for 5 s. For the preparation of an aqueous solution with acidic conditions, acetic acid was added to water and adjusted with hydrochloric acid. An alkaline aqueous solution was prepared by adding sodium bicarbonate to water, adjusted with sodium hydroxide. Washing solutions was prepared as following:

Washing solutions with pH of 2: Glacial acetic acid (2 ml) + distilled water (100 ml) +HCL (0.1 N, 2.5 ml).

Washing solutions with pH of 5: Glacial acetic acid (6 µl) + distilled water (100 ml).

Washing solutions with pH of 7: distilled water (100 ml).

Washing solutions with pH of 9: sodium bicarbonate (2 g) + distilled water (100 ml) + NaOH (1 N, 3 ml).

Washing solutions with pH of 9: sodium bicarbonate (2 g) + distilled water (100 ml) + NaOH (50% w/v, 2 ml).

### 2.5 The cooking process of *Agaricus bisporus* samples

The processing of microwaving, boiling, and frying were performed separately on each sample (50 g). In the microwave technique, samples were placed inside a microwave at 700 watts for 3, 5, and 7 min. In the boiling process, samples were immersed in 200 ml of water and boiled for 5, 10, and 15 min. For the surface frying, samples were placed in a pan containing 10 g of frying oil (180°C) for 3, 7, and 10 min. It should be noted that each experiment was performed in triplicate.

### 2.6 Extraction of pesticide

To extract the pesticides, the QuEChERS method (Quick, easy, cheap, effective, rugged, and safe sample preparation method) was carried out according to the procedure reported a by previous study after some modifications (Nasiri et al., 2016). Briefly, an aliquot of 50 μl of triphenylmethane solution (TPM, 1,000 mg/L) as the internal standard solution was added to 50 g of the *Agaricus bisporus* sample, which had been thoroughly ground using a mill. Then, 10 g of the ground sample was transferred to a falcon tube of 50 ml, and 40 µl of triphenylmethane (100 µg/kg) was added and stored for 30 min at 4°C. After adding 10 ml of acetonitrile, the sample was mixed in a vortex for 2 min. Then, 1 g of sodium chloride and 0.5 g of magnesium sulfate were added and stirred for 2 min. The samples were centrifuged (5,000 RPM for 10 min), and the supernatant was transferred to the falcon containing 1 g of magnesium sulfate and 0.5 g of PSA. The samples were then stirred for 2 min and centrifuged (5,000 RPM for 10 min) again. Four ml of centrifuged specimens were transferred to a falcon tube containing 40 μl of 5% formic acid and dried under nitrogen gas to a volume of 0.5 ml. Toluene was added to the dried residue to a final volume of 1 ml and stirred for 3 min. Finally, 2 μl was injected into the GC/MS.

### 2.7 Apparatus and chromatographic conditions

In this study, a gas chromatography/mass spectrometry (GC‐MS) of Agilent 6890N (Wilmington, USA) equipped to HP‐5 column (with a length of 30 m, inner diameter of 250 mm and a particle size of 0.25 µm) was used to measure pesticide residues. The temperatures of the ionizer, analyser and injector were 230, 150 and 250°C, respectively. Helium gas was used as the carrier gas. The initial temperature of the column was 120°C; the column remained at this temperature for 3 min and then reached a temperature of 180°C at a rate of 25°C/min before reaching a temperature of 300°C without stopping at a rate of 5°C/min. It remained for 10 min at this temperature.

### 2.8 Validation analysis method

Calibration curves for each pesticide were constructed using external standards ranging from 0.025 to 0.5 mg/kg, prepared by the dilution of a stock solution in ethyl acetate. The limits of detection (LOD) and limits of quantification (LOQ) were calculated as S/*N* = 3 and S/*N* = 9, respectively, where S/N is the ratio of signal/noise in a spiked matrix (Hassani et al., 2013). The reliability of the data of the pesticide analysis was determined by conducting internal quality control experiments, in addition to using validated methods. In this regard, recoveries of pesticides were recorded by analyzing *Agaricus bisporus* samples spiked with specific concentrations (0.01, 0.1, and 1 µg/kg) of each pesticide.

### 2.9 Calculation of process factor

To determine the reduction or condensation of each pesticide, process factors (PFs) were determined during storage, washing, and cooking. For this purpose, the concentration of pesticides in the processed sample was divided into its concentration in unprocessed samples. If the process factor was more than one, it meant that the concentration of the pesticide in the processed sample was more than in the unprocessed samples, while if the process factor was lower than one, it indicates the concentration of the pesticide in the processed sample was remarkably reduced (Pazzirota, Martin, Mezcua, Ferrer, & Fernandez‐Alba, 2013).

### 2.10 Statistical analysis

All experiments were performed in triplicate, and pesticide concentration was recorded as wet weight. After each processing, the reduction percentage for each pesticide was calculated. The total data were expressed as the mean ± standard deviation (*SD*) and analyzed by SPSS software, version 16.0. The analysis of variance (ANOVA) followed by a Tukey test was used to compare the reduction percentage of pesticides among different groups. A P‐value of less than 0.05 was considered statistically significant.

## 3 RESULTS AND DISCUSSION

The retention time, calibration data, diagnostic ions, and the selected quantification ion for the target pesticides analyzed by GC/MS are shown in Table 1.

**Table 1:** Retention times, calibration data, diagnostic ions, and the selected quantification ion for the pesticides

Compound	Retention time	Linear equation	Regression Coefficient	LOD^a^ (mg/kg)	LOQ^a^ (mg/kg)	Diagnostic ions	Quantification
Diazinon	18.28	y = 0.23874x + 0.1041	0.9936	0.005	0.015	179, 304, 267	304
Malathion	28.82	y = 0.23595x + 0.1071	0.9919	0.004	0.012	173.1, 158, 125	173.1
Permethrin	33.66	y = 0.05289x + 0.0139	0.9926	0.006	0.019	183.1, 165, 163.1, 264, 149	163.1
Propargite	27.63	y = 0.12506x + 0.0182	0.9914	0.007	0.023	350, 201.1, 135, 173.1	350
Fenpropathrin	30.53	y = 0.18334x ‐ 0.1753	0.9908	0.004	0.013	265.1, 208, 181.1	181.1

a LOD: limits of detection and LOQ: limit of quantitation.

The recovery percentage of diazinon, malathion, permethrin, propargite, and fenpropathrin ranged from 93.89%–104.42%, 94.02%–106.92%, 100.08%–108.95%, 96.64%–105.06%, and 86.14%–94.97%, respectively. These percentages were in an acceptable range according to EU guidelines (Sanco, 2011).

In the current study, the processing factor for all pesticides during studied methods was lower than 1, which indicated that the concentration of each pesticide after any processing was lower than its level in the raw sample (data not shown).

The average initial residues of diazinon, malathion, permethrin, propargite, and fenpropathrin in contaminated *Agaricus bisporus* samples were 1.86 ± 0.14 mg/kg, 0.71 ± 0.04 mg/kg, 1.58 ± 0.08 mg/kg, 1.04 ± 0.05 mg/kg, and 0.37 ± 0.02 mg/kg, respectively.

**Figure 1:**
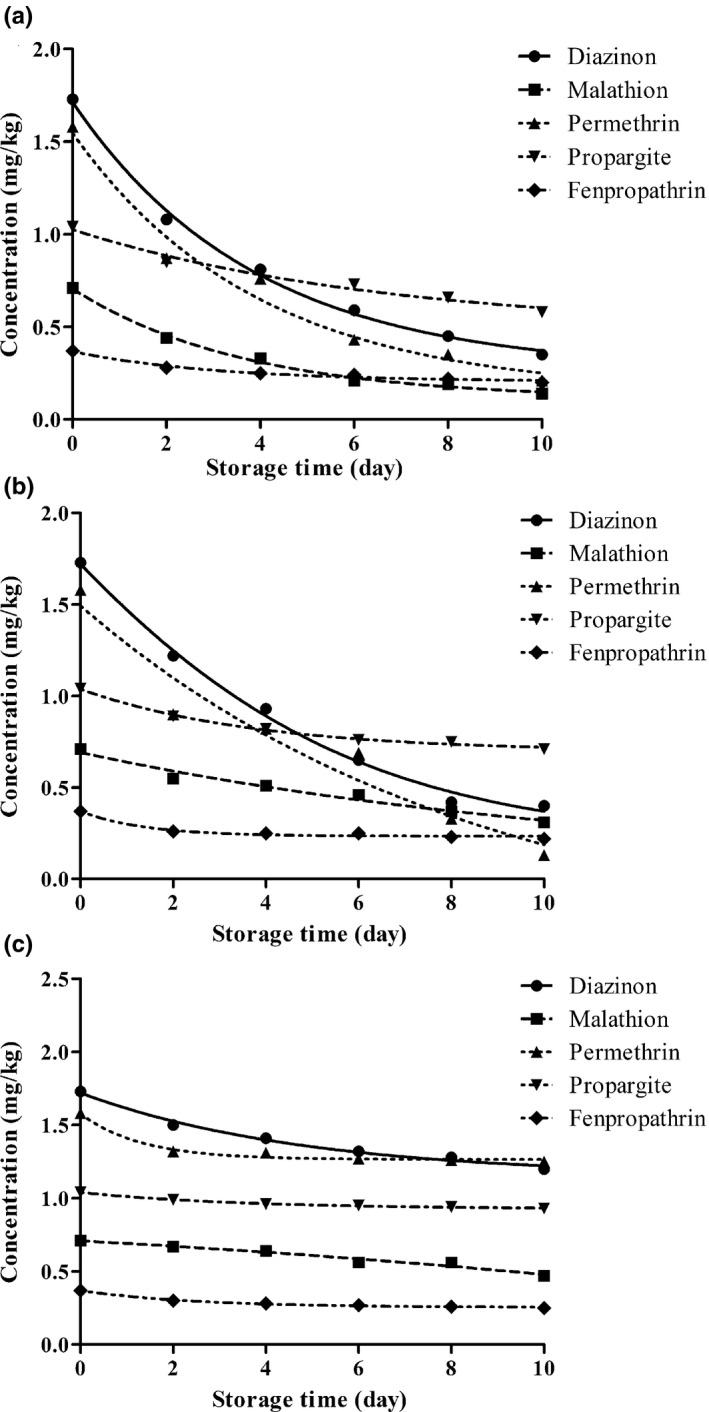
Dissipation kinetics of pesticides in Agaricus bisporus stored in room temperature (a), refrigerator temperature (b), and freeze temperature (C)

Figure 1 shows the concentration changes of each pesticide during storage at room (25°C), refrigerator (4°C), and freezer temperature (−18°C). The half‐life values (*t*_1/2_) and dissipation rate constants (k) of the five pesticides are shown in Table 2. At room temperature, the lowest and highest half‐life values were 4.44 days for diazinon and 13.08 days for propargite, respectively. At freezer temperatures, the lowest (20.39 days) and highest (69.31 days) half‐life values was related to diazinon and propargite, respectively.

**Table 2:** Equation of dissipation kinetics and half‐life of pesticides in *Agaricus bisporus* stored in various conditions

Storage condition	Diazinon	Malathion	Permethrin	Propargite	Fenpropathrin
Room temperature					
Equation of degradation kinetics	y = 1.5751e^−0.156x^	y = 0.6359e^−0.159x^	y = 1.4773e^−0.193x^	y = 0.9924e^−0.053x^	y = 0.3371e^−0.055x^
*R* ^2^	.9886	.9760	.9803	.9708	.9145
t_1/2_	4.44	4.36	3.59	13.08	12.60
Refrigerator temperature					
Equation of degradation kinetics	y = 1.6817e^−0.155x^	y = 0.6909e^−0.078x^	y = 1.7365e^−0.223x^	y = 0.9807e^−0.035x^	y = 0.3185e^−0.041x^
*R* ^2^	.98	.97	.86	.9104	.7078
t_1/2_	4.47	8.89	3.11	19.80	16.91
Freezer temperature					
Equation of degradation kinetics	y = 1.6542e^−0.034x^	y = 0.7263e^−0.04x^	y = 1.462e^−0.019x^	y = 1.0182e^−0.01x^	y = 0.3391e^−0.034x^
*R* ^2^	.9466	.9504	.6686	.91	.83
t_1/2_	20.39	17.33	36.48	69.31	20.39

The findings indicated that the dissipation rate of acaricides (propargite and fenpropathrin) was lower than that of insecticides such as diazinon, malathion, and permethrin. This difference in rate could be due to the structural differences in these pesticides. Propargite and fenpropathrin are pyrethroid pesticides, while diazinon, malathion, and permethrin are categorized as organophosphorus compound groups. In addition, the volatility and octanol‐water partition coefficient influenced on storage stability of the pesticides, and the presence of a thioether bond in some pesticides, such as malathion, caused them to be unstable (Bian, Liu, Chen, & Sun, 2018).

Temperature was one of the most important factors in storage stability of pesticides. A remarkable loss of the residues occurred over time, even at low temperatures (Bian et al., 2018). In the current study, the findings were different that other results reported in the literature. In this study, 50% of diazinon and malathion remained for 20.39 and 17.33 days, respectively, during storage in freezer temperatures, while Bian et al. (2018) reported 75% of diazinon and 49% of malathion residue in a cucumber sample after 180 days of storage in −20°C. Cengiz, Certel, and Göçmen (2006) found that diazinon residue in a cucumber was reduced 36% after 3 days and 65% after 6 days at 4°C, respectively. In this study, the dissipation rate of malathion at room temperature (4.36 days) was more rapid than that of barley samples reported by Kong et al. (2016), who found that more than 50% of malathion was dissipated within 7 days of storage. The discrepancy between results may be explained by a difference between matrix properties in various plants. For instance, the studies showed that a catalase enzyme could increase the breakdown of pesticide residue in a cucumber sample; therefore, it decreased the half‐life value of pesticides (Bian et al., 2018).

For assessment of the impact of acid or alkaline conditions, the reduction of different pesticides was determined in a pH of 2–12. In general, the highest reduction of diazinon, malathion, permethrin, propargite, and fenpropathrin was found in pH levels of 12, 2, 12, 7, and 9, respectively (Table 3). In most cases, a significant difference did not exist among the mean reduction of pesticides during washing with various pH solutions at certain washing times.

**Table 3:** Pesticide reduction percentage in *Agaricus bisporus* during washing process with various pH solutions

Pesticide name	Washing time (min)	Water solution with various pH				
		2	5	7	9	12
Diazinon	10	65.90 ± 1.73^Dc^	68.98 ± 1.85^Dcb^	70.14 ± 2.84^Cb^	71.89 ± 6.21^Ab^	77.51 ± 3.02^ABa^
	20	74.76 ± 0.88^Ca^	72.83 ± 2.08^CDa^	73.41 ± 2.01^Ca^	77.32 ± 2.68^Aa^	76.50 ± 4.43^Ba^
	30	81.12 ± 3.93^Ba^	77.26 ± 1.76^BCa^	79.77 ± 1.52^Ba^	79.71 ± 2.11^Aa^	82.01 ± 1.65^ABa^
Malathion	10	74.65 ± 1.41^Cab^	79.81 ± 2.82^Aa^	72.77 ± 3.54^Cb^	74.65 ± 6.14^Aab^	80.75 ± 3.54^ABa^
	20	81.22 ± 2.15^Ba^	80.28 ± 2.15^Aa^	79.34 ± 2.15^ABa^	79.81 ± 2.9^Aa^	79.81 ± 4.30^ABa^
	30	91.08 ± 3.25^Aa^	84.04 ± 1.62^Aab^	80.28 ± 8.57^Ab^	82.16 ± 2.15^Ab^	84.98 ± 1.62^Aab^
Permethrin	10	38.76 ± 1.25^Fb^	42.50 ± 1.90^Eb^	42.85 ± 1.55^Fb^	42.85 ± 1.55^BCb^	54.31 ± 4.75^Da^
	20	47.3 ± 1.52^Fb^	45.71 ± 2.05^Eb^	46.80 ± 2.33^Fb^	46.17 ± 1.95^BCb^	61.41 ± 1.26^CDa^
	30	49.99 ± 1.27^Fb^	47.38 ± 1.27^Eb^	48.61 ± 1.89^Fb^	47.76 ± 1.48^Bb^	66.46 ± 1.25^Ca^
Propargite	10	27.56 ± 2.41^HGb^	27.56 ± 0.55^Fb^	56.41 ± 2.41^Ea^	28.2 ± 1.47^Db^	30.63 ± 2.01^Gb^
	20	45.83 ± 2.54^Ec^	43.59 ± 1.46^Ebc^	62.50 ± 3.46^Da^	41.03 ± 3.09^Cc^	41.67 ± 3.88^Fc^
	30	49.04 ± 1.46^Ebc^	44.23 ± 5.08^Ec^	68.27 ± 3.47^DEa^	48.72 ± 1.46^Bbc^	51.92 ± 3.84^Eb^
Fenpropathrin	10	28.82 ± 4.12^Gab^	17.11 ± 6.80^Gb^	33.33 ± 6.80^Ga^	36.03 ± 10.23^CDa^	28.82 ± 4.13^Gab^
	20	26.13 ± 8.25^GHa^	17.12 ± 9.49^Ga^	31.53 ± 1.56^Ga^	30.63 ± 13.87^Da^	25.12 ± 10.92^GF^
	30	22.524 ± 4.12^Hb^	32.43 ± 2.71^Fa^	33.33 ± 1.56^Ga^	33.33 ± 5.62^CDa^	30.63 ± 4.12^Ga^

Note

Means not sharing common superscript capital and small letters are significantly different within the same column and row, respectively (*p* < .05). One‐way ANOVA followed by Duncan's test was applied to determine the different among groups (each mean was obtained from three different tests).

Overall, the effectiveness of the washing solution in reducing pesticide contamination could be related to the difference between the pH solution and the PK_a_ value of the pesticides. The PK_a_ value of a given molecule defines the pH at which it is neutral. Generally, an acidic molecule would be charged in basic condition, while it would be uncharged in an acidic condition. According to the Henderson–Hasselbalch equation, depending on the difference between the PK_a_ value of a molecule and the pH, the ratio of ionized molecules to non‐ionized molecules is different (Reijenga, Hoof, Loon, & Teunissen, 2013). The number and distribution of charges on a molecule affect its aqueous solubility. Therefore, in this study, diazinon (PK_a_ = 2.8) was expected to be better rinsed with a solution of a higher pH (pH = 12) than other pesticides. In contrast, malathion, as a basic agent, was better rinsed through a washing solution with a lower pH (pH = 2).

In the present study, washing with plain water (pH = 7) had a different impact on the pesticides. The order of reduction was as follows: malathion > diazinon>propargite > permethrin>fenpropathrin. The amount of reduction may have been related to the level of the pesticide's solubility in an aquatic condition. In water at 25°C, the solubility of diazinon and malathion was higher than 50 mg/L, while other pesticides such as permethrin, propargite, and fenpropathrin had a solubility lower than 1 mg/L.

In this study, a sodium chloride solution with logarithmic concentrations was used to wash *Agaricus bisporus*. The results indicated that the reduction of different pesticides did not depend greatly on the chloride sodium concentration (Table 4). However, at each level of chloride sodium, with increased washing time of 10–30 min, the pesticide reduction was raised. Contrary to these findings, Randhawa et al. (2016) showed that a concentration of 10% sodium chloride for 10 min reduced imidacloprid content from 1.170 to 0.649 mg/kg (44.52%), and acetamaprid from 1.240 to 0.606 mg/kg (51.12%). Rasolonjatovo et al. (2017) reported that, during washing of a tomato sample with a sodium chloride (5%) solution for 50 min, methomyl and acetamiprid residues were reduced 49% and 47%, respectively. Depending on the concentration of salt, organic compounds such as pesticides exhibit different solubility in the aqueous medium (Rasolonjatovo et al., 2017). On the one hand, Alister et al. (2018) declared that sodium chloride solution is a strong electrolyte, which has a charge that can interact with the pesticides and create an attractive force to ensure their removal. On the other hand, high salt concentrations cause organic compounds to precipitate in an aqueous medium through the salting‐out phenomenon. In the present study, the salt level could not make a significant difference in the solubility of studied pesticides.

**Table 4:** Pesticide reduction percentages in *Agaricus bisporus* during washing process with different concentrations of sodium chloride

Pesticide name	Washing time (min)	Sodium chloride concentration (%w/v)			
		0	0.1	1	10
Diazinon	10	69.36 ± 1.15^Ca^	71.29 ± 0.88^a^	71.29 ± 0.88^Ca^	70.14 ± 2.84^Ca^
	20	74.57 ± 0.57^Ba^	70.91 ± 3.18^a^	70.9 ± 2.19^Ca^	73.41 ± 2.01^ABCa^
	30	77.07 ± 1.45^Ba^	77.07 ± 1.45^a^	77.07 ± 3.18^BCa^	79.77 ± 1.52^ABa^
Malathion	10	76.53 ± 2.14^Bab^	81.22 ± 1.75^Ba^	72.3 ± 1.09^Cb^	72.77 ± 3.54^BCb^
	20	82.16 ± 2.15^Aa^	81.22 ± 2.89^Ba^	79.34 ± 2.16^Ba^	79.34 ± 1.95^ABa^
	30	84.04 ± 2.15^Ab^	91.08 ± 3.01^Aa^	85.45 ± 2.06^Aab^	80.28 ± 8.57^Ab^
Permethrin	10	42.85 ± 1.55^Gab^	38.85 ± 1.26^EFb^	34.06 ± 3.49^Ec^	46.38 ± 2.27^Ea^
	20	46.8 ± 2.33^FGb^	43.73 ± 1.57^DEcb^	41.75 ± 1.29^Dc^	53.06 ± 2.05^Ea^
	30	48.61 ± 1.89^Fb^	47.51±0.59^Db^	46.17 ± 1.95^Db^	61.30 ± 1.83^Da^
Propargite	10	56.41 ± 2.41^Ea^	32.37 ± 4.33^Gc^	32.37 ± 1.46^Ec^	49.04 ± 2.53^Eb^
	20	62.5 ± 3.46^Da^	38.46 ± 1.66^Fb^	40.06 ± 5.29^Eb^	60.58 ± 1.92^Da^
	30	68.27 ± 3.47^Ca^	42.63 ± 3.37^EFb^	45.51 ± 3.95^Db^	68.91 ± 1.46^Ca^
Fenpropathrin	10	33.33 ± 6.81^Ha^	22.52 ± 5.62^Ga^	31.53 ± 8.25^Ea^	25.22 ± 6.75^Ga^
	20	31.53 ± 1.55^Ha^	33.34 ± 4.12^Ha^	29.73 ± 2.71^Ea^	29.73 ± 2.71^FGa^
	30	33.33 ± 1.56^Ha^	32.43 ± 2.70^Ga^	30.63 ± 4.13^Ea^	32.43 ± 7.15^Fa^

Note

Means not sharing common superscript capital and small letters are significantly different within the same column and row, respectively (*p* < .05). One‐way ANOVA followed by Duncan's test was applied to determine the different among groups (each mean was obtained from three different tests).

The effect of the three methods—boiling, frying, and microwaving—on the reduction of various pesticides is shown in Table 5. Regardless of the method of cooking, a significant decrease was observed in the level of pesticides with an increase in the duration of cooking. The cooking process used had different impacts on the pesticide. The order of the average pesticide decrease was as follows: permethrin > malathion>diazinon > fenpropathrin>propargite. The stability of organophosphates, such as permethrin, malathion, and diazinon, was lower than that of propargite and fenpropathrin in various techniques.

**Table 5:** Pesticide reduction percentage in *Agaricus bisporus* during various cooking methods

Pesticide name	Cooking mode									Average reduction			
	Boiling			Frying			Microwave			Boiling	Frying	Microwave	*p* Value
	Boiling time (min)			Frying time (min)			Microwave time (min)						
	5	10	15	5	7	10	5	7	10				
Diazinon	44.32 ± 1.45^Cf^	57.99 ± 1.20^cBd^	73.22 ± 0.88^ABb^	49.9 ± 1.21^Bef^	60.5 ± 1.20^Cc^	70.52 ± 2.08^BCb^	51.64 ± 2.61^Bde^	69.36 ± 1.15^Ab^	79.96 ± 10.69^Aa^	58.50	60.30	66.98	.165
Malathion	52.58 ± 2.92^ABde^	62.91 ± 1.62^ABc^	67.14 ± 3.54^BCbc^	51.17 ± 0.81^Be^	65.26 ± 4.07^Bbc^	74.65 ± 1.41^ABa^	55.87 ± 1.62^BCd^	68.08 ± 2.92^Ab^	78.87 ± 1.41^Aa^	60.87	63.69	67.60	.160
Permethrin	58.4 ± 2.27^Ae^	68.99 ± 2.28^Ad^	75.95 ± 1.68^Ac^	59.53 ± 2.72^Ae^	78.69 ± 0.96^Ab^	83.54 ± 3.84^Aa^	59.61 ± 0.48^Ae^	72.57 ± 1.31^Ac^	83.33 ± 0.97^Aa^	67.77	73.92	71.83	.226
Propargite	24.04 ± 1.46^Dd^	49.04 ± 1.99^Cb^	61.54 ± 1.92^Ca^	30.77 ± 2.42^Dc^	50.96 ± 1.66^Db^	62.5 ± 1.99^Ba^	33.65 ± 2.42^Dc^	52.88 ± 2.93^Bb^	64.42 ± 2.94^Ba^	44.87	47.86	50.42	.459
Fenpropathrin	45.95 ± 8.25^BCa^	45.95 ± 9.75^Ca^	48.65 ± 8.25^Da^	40.54 ± 4.12^Ba^	43.24 ± 2.71^Ea^	45.95 ± 4.05^Da^	45.95 ± 4.12^Ca^	48.65 ± 7.14^Ba^	48.65 ± 7.15^Ca^	46.86	43.24	47.44	.219

Note

Means not sharing common superscript capital and small letters are significantly different within the same column and row, respectively (*p* < .05). One‐way ANOVA followed by Duncan's test was applied to determine the different among groups (each mean was obtained from three different tests).

Although information regarding the impact of cooking on permethrin, propargite, and fenpropathrin residue is rare, various reports are available in existing literature regarding the role of the cooking process on the residue of organophosphates such as diazinon and malathion in foods. In the current study, the mean diazinon reduction during boiling, frying, and microwaving was found to be 58.5, 60.3, and 66.98%, respectively, and no significant difference was observed between the reduction recorded during three methods. The reduction level obtained in our study was lower than that reported by Kang and Lee (2005) in cabbage samples (80%–90%) boiled for 30 min. In agreement with our data, Lalah and Wandiga (2002) found that cooking with and without sodium chloride could reduce the malathion level in maize grains up to 56.7 and 69.7%, respectively. This reduction level was reported as 64.2 and 75% for malathion in beans in the presence and absence of salt, respectively. Various causes behind pesticide reduction during cooking were presented. Alister et al. (2018) found that insecticide reduction was correlated with aqueous hydrolysis, molecular mass, boiling point, Henry constant, and the organic carbon soil adsorption coefficient. During heat processing, including boiling, blanching, cooking, pasteurization, and frying, the pesticide residue was reduced, possibly due to degradation, co‐distillation, or evaporation. However, the magnitude of pesticide residue dissipation depended on the physicochemical characteristics of pesticide, the matrix type, and the mode of heating (Alister et al., 2018; Bian et al., 2018).

## 4 CONCLUSIONS

The current study investigated the effect of storage conditions and washing methods, as well as the various cooking processes, on residue levels of five common pesticides—diazinon, malathion, permethrin, propargite, and fenpropathrin—in *Agaricus bisporus* products. The results showed that the effectiveness of the washing solution in reducing pesticide contamination was related to the difference between the pH solution and the PKa value of the pesticides. The effect of various salt concentrations on the washing process showed that a reduction in the studied pesticides was not related to the salt level in the washing solution. Although there was no significant difference in the average reduction of each pesticide between the various cooking processes, the cooking time was an important factor in the removal of pesticides. Therefore, it can be concluded that the use of washing solutions with the appropriate pH as well as increased fungal cooking time can be useful in reducing the risks of exposure to the studied pesticides.

## CONFLICT OF INTEREST

Authors have no conflict of interests.

## ETHICAL APPROVAL

This study does not involve any human or animal testing.
